# Compression of The Sciatic Nerve May not Contribute to Ipsilateral Hyperalgesia Development in Ovariectomized Female Rats!

**DOI:** 10.22074/cellj.2021.6907

**Published:** 2020-04-22

**Authors:** Nafissa Telailia, Sylvain Fisson, Hacène Frih

**Affiliations:** 1 Department of Biology, Faculty of Natural Science, University of Badji Mokhtar, Annaba, Algeria; 2 National Institute of Health and Medical Research, Joint Research Unit_S951, Evry, France; 3 Genethon, National Institute of Health and Medical Research, Joint Research Unit_S951, Evry, France; 4 Joint Research Unit_S951, University of Evry Val d'Essonne, Evry, France

**Keywords:** Filaments, Injury, Mechanical Allodynia, Mechanical Hyperalgesia Neuropathic Pain

## Abstract

**Objective:**

von Frey Filament (vFF) is an aesthesiometer to measure paw withdrawal thresholds. Our aim was to validate the manually von Frey test technique for assessing neuropathic pain behavioral signs in a sciatic nerve ligation model.

**Materials and Methods:**

In this experimental study, peripheral neuropathic pain associated with sciatic nerve chronic ligation (SN-CL) was induced. Filaments used against posterior pad mid-plantar region using a simplified up-down method (SUDO). In addition to baseline withdrawal thresholds, the behavioral test was repeated after surgery thrice more with an interval of ten days. vFF (2 to 26 g) were used in ascending order for hyperalgesia assessment.

**Results:**

In SN-CL rats, the results validate a loss of pain sensation, resulted in, long-lasting ipsilateral allodynia with the development of contralateral allodynia later and an extraterritorial development of neuropathic signs. Variability for the development of ipsilateral and contralateral allodynia over time was noted in sham (SH) control rats. SN-CL group showed a contralateral hyperalgesia development just at the 16th-day after surgery with an absence of ipsilateral hyperalgesia development at the different days of paw withdrawal thresholds measurements.

**Conclusion:**

Manually vFF test technique was successfully used for assessing neuropathic pain behavioral signs in sciatic a nerve ligation model with the absence of ipsilateral hyperalgesia development.

## Introduction

Neurological disorders may lead, directly or indirectly, to pain with physiological and physical dimensions that are both essential for its diagnosis and treatment. Daily, pain sensation specifically is evoked by potential or actual noxious (i.e., tissue-damaging) stimuli applied to the body that differs from pain during disease that occurs in the absence of external noxious stimuli (1). “Pain is a mutually recognizable somatic experience that reflects a person’s apprehension of threat to their bodily or existential integrity” (2). This definition was qualified by the Taxonomy Task Force of the association in 1994 (3) "Pain is always subjective. Each individual learns the applications of the word through experiences relating to injuries in early life" (4).

There are three types of pain: acute physiological nociceptive pain, pathophysiological nociceptive pain, also called hyperalgesia and/or allodynia. Hyperalgesia is an extreme pain intensity felt upon noxious stimulation, and allodynia is the sensation of pain elicited by stimuli that are normally below the pain threshold. The sensory system itself can be damaged and become the source of continuous pain. This type of pain is classified as neuropathic. Chronic neuropathic pain has no physical protective role as it continues without obvious ongoing tissue damage. In contrast to nociceptive pain, which is the result of stimulation of primary sensory nerves for pain, neuropathic pain occurs when a lesion or disruption of function occurs in the nervous system. If the cause is located in the central nervous system (CNS) (brain or spinal cord) it gives rise to central neuropathic pain, and if it is located in the peripheral nervous system, it gives rise to peripheral neuropathic pain. Neuropathic pain may be induced by physical injuries of the nervous system, such as surgery (5). Murine models of peripheral nerve injury often target the sciatic nerve which is easy to access, produces behavioral signs of neuropathic pain, including mechanical allodynia (pain perception upon the innocuous tactile stimuli) and hyperalgesia (exaggerated pain sensations by mildly noxious stimuli). Sciatic nerve chronic ligation (SN-CL) allows nociceptive tests on the hind paws that end by studying of neuropathic pain mechanism, pain sensory and for the study of neuropathic pain, treatments using von Frey filaments (vFFs) test. For this aim, sciatic nerve ligation was a target which induces long-lasting mechanical allodynia (6, 7). This last was measured by using vFFs, a highly sensitive test in detecting allodynia in conditions likely to cause neuropathic pain, and as soon as allodynia is established in the animal, it is easily quantified (8) Von Frey test is a pressure test to detect the perception of light touch. It involves the use of a thin and flexible filament applied to the skin with just enough force to induce a bend in the filament (9). For best comparisons, prior sciatic nerve ligation, we established baselines thresholds values that represent the normal and the beginning thresholds level of nociceptive measurements.

Mechanical allodynia was assessed with the Von Frey test, von Frey monofilaments were utilized for the estimation of paw withdrawal threshold in both hind paws as a measure of mechanical allodynia in our peripheral neuropathic pain model. Hind paw withdrawal thresholds were determined as a measure of ipsilateral and contralateral mechanosensitivity for chronic sciatic nerve ligation (SN-CL) and sham (SH) female rats by the Simplified Up-and-Down Order (SUDO) method, which is more recently developed.

Studying behavioral signs of neuropathic pain in sciatic nerve ligation model compared with SH rats (animal underwent the same surgical procedures without seeing the sciatic nerve ligation) using vFF test technique manually at different days post-surgery was the aim of this study.

## Materials and Methods

### Biological material

In this experimental study, female rats White Wistar Rats from the Pasteur Institute of Algiers were used in our study as the biological material for the reason that female are more sensitive to pain than males because of ovarian hormones (10). Under the environmental conditions of the experiment room (natural photoperiod, humidity, temperature, etc.), the animals were kept in polyethylene cages (8 female rats per cage), given 20 g of food per female rat daily with ad libitum access to water.

After an adaptation period of four weeks, 16 females aged 3 months approximately were randomly selected, they were with an average weight of 214.4 ± 6.389 g and divided into two experimental groups (eight females per group): the rates of the first group were underwent ligation of sciatic nerve in the left hind paws, the second group consists of SH rats (the animal underwent the same surgical procedures without seeing the sciatic nerve ligation in that paws). Contralateral hind paws were always intact in the two groups. Rats weighted one day prior to each test and five days after surgery to determine antibiotic doses.

All applicable international, national, and/or institutional guidelines for the care and use of animals were followed (D01N01UN230120150001).

### Surgery procedure for sciatic nerve ligation

#### Anesthesia

Rats were anesthetized by intraperitoneal injection of ketamine hydrochloride (5 mg∕Kg i.p., ketamine)+a drop orally of chlorpromazine, and an ophthalmic ointment was applied to the eyes of animals using a cotton swab to avoid increased intra-ocular pressure (11) caused by ketamine. The animals were placed in a calm and quiet place until fully anesthetized. The reflex of rats was checked by pinching the tip of the tail and legs with a pair of tweezers to ensure the immobility of animals before any surgery.

#### Surgery

According to (12) chronic constriction injury model, surgical area (using an electric razor) was shaved. The animal was placed on its right side, and the left hind limb was put on a small platform in order to keep it high. The leg was fixed with tape. The operative field was disinfected with alternating scrubs of ethanol and betadine outside the surgical site. The knee was located with the thumb of the left hand, and a scalpel was used to make an incision of few centimeters (cm) in the proximal longitudinal direction of the knee. Then, the skin was opened by blunt dissection using the tip of a pair of sterilized scissors. The muscular layer was separated by dissection just next to the visible blood vessel, and we closed the femur (thigh bone). The muscle layers were easily separated without bleeding and then left sciatic nerve below the muscle was revealed. The rat was placed under a stereomicroscope to gently separate the muscles with a pair of tweezers sterilized to visualize the sciatic nerve. The area and the collateral saphenous branches of the sciatic nerve were identified, knowing that the sural nerve is the smallest of the three branches and three tight surgical knots were created around the sciatic nerve. When the first node was done, a contraction of paw’s muscle groups supplied by the sciatic nerve that underwent ligation was observed. After that, the suture ends were cut with a pair of micro-scissors and the muscle layer were gently closed. Finally, a drop of lidocaine was added on the wound, and we sutured with surgical knots. The SH control animals were anesthetized, skin and muscles were cut to expose the nerve similar to the ligated animals (except for the ligation). The skin was sutured back to close the opened tissue. For the two groups, contralateral hind paws were intact. SN-CL and sham-operated animals were allowed to recover for five days before testing in their ipsilateral and contralateral hind paws.

#### Post-surgery period

The sufficiency of eye ointment was checked. Then, the rat was placed in a clean cage under a paper towel in a comfortable posture near to a heat source. Water and food were easily accessible for the animal operated. Finally, intraperitoneal injection of 75000 IU∕Kg∕day of benzylpenicillin sodium (Algeria, Saidal, PEN G Panpharma) for five days after surgery was done.

#### Nociceptive behavioral test: Von Frey filaments test

##### Sensory evaluators (Semmes-Weinstein Monofilaments)

In our study, standard esthesiometers set which contains 20 nylon monofilaments have been used to assess mechanical sensitivity. vFFs that are soft nylon hairs of different lengths and calibrated diameters to known forces, providing discrete units of pressure and fixed on hand-held applicators (13) were used to estimate hind paw withdrawal thresholds for SN-CL and SH female rats. The enabling principle of the Von Frey hair methodology for assessing skin sensitivity to crude touch is that a hair (or a plastic monofilament) will exert increasing pressure on the skin as it is pressed harder and harder (14) before the filament starts to bend. However, after bending, the vertical force was constant. The force was directly proportional to the stiffness, directly related to the thickness of the filament and inversely proportional to its squared length (15, 16). Prior to the second and the third tests, two (2) minutes were sufficient for bending filaments to be automatically calibrated and to be straight.

The behavioral assessment was conducted by the same person, time, location, and it was performed in all animals before surgery (day 0), on days 6, 16 and 26 after SN-CL surgery or SH operation for the long assessment of neuropathic pain behavioral signs. The day of surgery was referred to as day1. The testing chamber consisted of an (85×35×35) cm transparent plastic box with wire mesh platform (0.5×0.5 cm grid size) allowing access to the plantar surface of the hind paws, and it was positioned over a support to keep it elevated and to visualize the testing area (the midplantar surface just posterior to the footpads: This area is innervated mostly by terminal branches of the sciatic nerve). Each rat was placed in the testing chamber and allowed to acclimate for 15 minutes prior to testing or as soon as the rat stopped exploring and appeared acclimatized to the testing environment and the Von Frey hairs were inserted through the mesh to poke the animal’s mid-plantar surface of hind paws, notice that the rat was tested when it was standing quietly on all four paws and was unaware of the experimenter’s hand.

##### Assessment of temporal evolution of tactile paws withdrawal thresholds in sciatic nerve chronic ligation and sham rats

The values of tactical paw withdrawal thresholds in SN-CL and SH rats was performed at basal (day 0), at the 6^th^, 16^th^, and 26^th^ days after surgeries in ipsilateral and contralateral hind paws for both SN-CL and SH control rats.

##### Measurement of mechanical hind paws withdrawal threshold by the SUDO method

The hind paw withdrawal threshold was determined using vFFs and was expressed in Millinewton (mN) by the SUDO method which requires an empirically determined filament force range and starts at the midrange filament for five consecutive touches (17). In the current study, to assess rat hind paw mechanical allodynia thresholds for SN-CL and sham-operated rats, we started with the mid-range filament (2.0 g). The filament was applied at a 90º angle for 2 seconds to the mid-plantar surface just posterior to the footpads of the left hind paw until it was just bent and then withdrawn, the procedure was repeated five consecutive times with an interval of 2 seconds between each stimulation. The response was considered positive if at least three expected responses were observed out of five applications. The expected responses were: Paw withdrawal, sudden flinching, and licking/biting of the stimulated paw. The response was recorded as an “X” for a positive response or an “O” if a negative response was observed.

If a positive response was reached on the first examined filament, then the next lowest filament was chosen. If no response was noted, then the next higher filament was used. This process was repeated until the first transition from the positive response to negative one –or vice versa– was obtained, after which force, was applied to the animal an additional four times following the up and down paradigm. If the last filament caused a response, a set value (0.5) was added to the filament force, whereas if no response was noted, the same value was subtracted from that filament force and was designated as SUDO result.

The force was determined from the newly designated SUDO result:

Filament force (mN)=0.0016×exp [(2.184 (-0.012 (SUDO result)^2^+0.429 (SUDO result)+1.359)].

Once the threshold was determined for the left hind paw, the same testing procedure was repeated on the right hind paw. The baseline withdrawal thresholds of each of the hind paws using von Frey hairs were determined for each rat prior to surgical manipulation (day 0). To assess the long-lasting mechanical allodynia, i.e., a nociceptive response to a normally non-nociceptive stimulus, measurement of the paw withdrawal threshold was repeated next on days 6, 16 and then on day 26 after SN-CL surgery or SH operation. The day of surgery was referred to as day 1.

#### Mechanical hyperalgesia assessment

For hyperalgesia assessment: 2, 4, 6, 8, 10, 15, and 26 g of vFFs were used until a filament induced a positive response (18). If no filament elicited a response, then the highest magnitude filament (26 g) was recorded as the threshold.

#### Data analysis

Graphs were plotted using Graphpad Prism version 7. Statistical analyses were conducted with Past3. The mean hind paw withdrawal thresholds were analyzed using one-way ANOVA (Kruskal Wallis for more than 2 times, in one group between different times and Mann Whitney for 2 times. Student’s t test, Fisher’s exact test, D-test, and Chi ²-tests were used for comparisons between or within groups of rats following one way ANOVA. The mean (± standard error of the mean) hind paw withdrawal thresholds values between different groups of rats or within the same group at different time points were considered significantly different with a P<0.05. For mechanical hyperalgesia assessment, graphs were plotted, and statistical analyses were conducted with the Graph-Pad Prism software version 6.

## Results

### Assessment of temporal evolution of tactile paws withdrawal thresholds in sciatic nerve chronic ligation and sham rats

**Fig.1:**
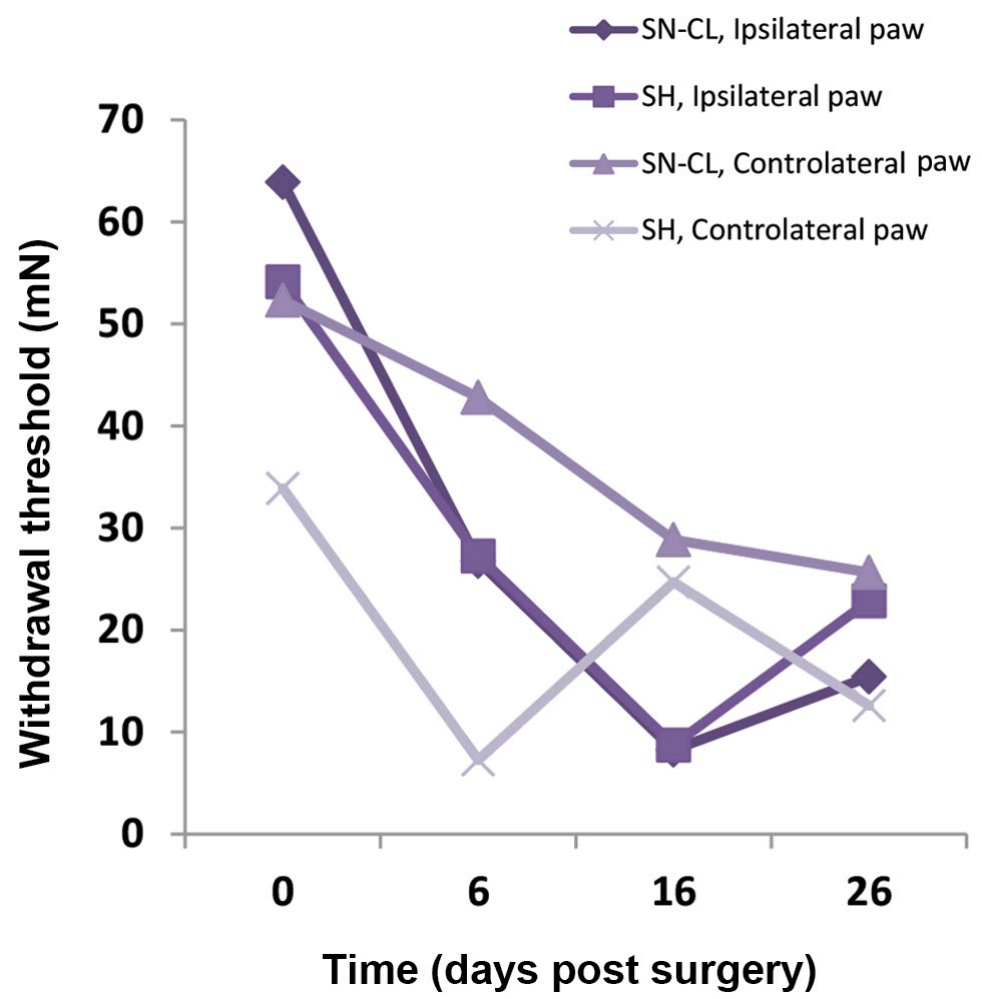
Temporal evolution of ipsilateral and contralateral mechanical paw withdrawal thresholds in sciatic nerve chronic ligation and sham groups (n=8).

The values of ipsilateral paw withdrawal thresholds of SNCL rats are almost identical as illustrated in SH animals from day 6 until day 16 post-surgery (Fig.1). After sixteen -days, the paw withdrawal response in SN-CL remains reduced until twenty -six days when compared with the SH group. For contralateral paw withdrawal thresholds, in SH rats are maintained reduced when compared with SN-CL ones from the 6^th^ to 26^th^ day post-surgery (Fig.1, Table 1).

**Table 1:** Sham animals time course of hind paws withdrawal thresholds mean in basal (day 0), at 6^th^, 16^th^, and 26^th^ days after surgery


Time (days)	Ipsilateral hind paws withdrawal thresholds mean (mN)	Contralateral hind paws withdrawal thresholds mean (mN)

0	54.13 ± 15.6	33.87 ± 12.64
6	20.45 ± 14.28	19.99 ± 14.25
16	6.548 ± 4.385	21.6 ±14.16
26	22.87 ± 13.84	12.59 ± 4.976


Data are presented as mean ± SE.

### Sciatic nerve chronic ligation rats time course of hind paws withdrawal thresholds mean in basal (day 0), at 6^th^, 16^th^ and 26^th^ days after surgery

In SN-CL rats, the ipsilateral mean paw withdrawal thresholds were reduced in the injured (ipsilateral) hind paw compared to the uninjured (contralateral) hind paw from few days after surgery to the last day of mechanical allodynia’s assessment, they were 20.06 ± 14.24, 7.22 ± 4.429, 13.50 ± 4.91 and 37.44 ± 17.65, 25.24 ± 3.985 and 19.22 ± 12.03 mN at the 6th, 16th, and 26th days after surgery, respectively in the ipsilateral paw. In contrast, the mean paw withdrawal thresholds the contralateral one were 7.22 ± 4.429, 37.44 ± 17.65, 19.22 ± 12.03 mM.

Data showed that ipsilateral paw withdrawal thresholds of nociceptive sensitivity were gradually lowered from six until the 16th -day post-surgery, they were varied from 63.91 ± 17 on day 0 to 13.50 ± 4.91 mN in the 16th-day post-surgery. In contrast, the mean withdrawal thresholds were gradually increased to 25.24 ± 3.985 on day 26 after surgery. This variability over time reached the significance from day 6 compared with the pre-operation (day 0) until day 26 (D-test P=0.0097 at 06 day, t test P=0.006 at 16 day, F-test P=0.004 at 26 day).

**Fig.2:**
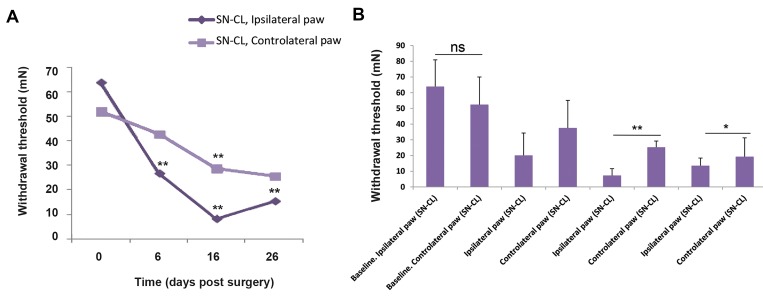
Repeated measurement of- ipsilateral and contralateral mechanical paw withdrawal threshold at 6th, 16th, and 26th days post- surgery in sciatic nerve chronic ligation rats (n=8). Asterisks indicate the significant difference in paw withdrawal threshold. ns; Non significant, *; P<0.05 , and **; P<0.005.

For contralateral nociceptive sensitivity, the mean paw withdrawal thresholds were reduced over time, they were varied from 52.32 ± 17.66 on day 0 to 19.22 ± 12.03 mN on the 26st day post-surgery. This decrease reached the significance from the 16th day compared with the preoperative (day 0) until the 26^th^ day (t-test P=0.56 at 06 day, F-test P=0.00084 at 16^th^ day, chi ²-test P=0.045 at the 26th day) (Fig.2A).

The analysis of tactile withdrawal threshold using oneway ANOVA, lead to a loss of a significant difference on day 0 (t test P=0.643) and at the 6^th^ after surgery (t test, P=0.45). In contrast, a statistically significant effect of surgery (sciatic nerve ligation) was revealed at the 16th -and 26^th^ days after surgery using Student’s t test (P=0.009) and Fisher’s exact test (F=6.0081, P=0.03) respectively (Fig.2B).

### Assessment of mechanical ipsilateral allodynia in sciatic nerve chronic ligation group in six, sixteen, and twenty-six-day post-surgery

**Fig.3:**
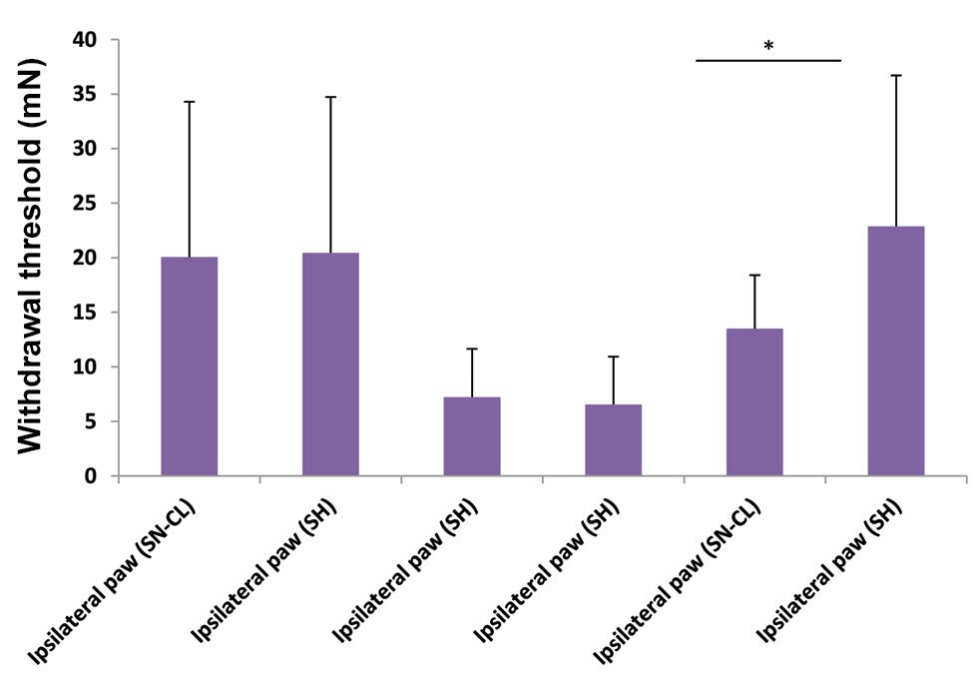
Comparing tactile ipsilateral paw withdrawal threshold of sciatic nerve chronic ligation (SN-CL) rats with sham (SH) group (n=8). Asterisks indicate the significant difference in paw withdrawal threshold. *P<0.05.

At six and sixteen days post-surgery, ipsilateral tactile withdrawal threshold in SN-CL was not significantly different from the SH group (t test, P=0.9). While, at the 26^th^ day after surgery, the mechanical sensitivity was decreased significantly when analyzed by the Fisher’s exact test (F=7.94, P=0.01, Fig.3).

### Mechanical contralateral allodynia in sciatic nerve chronic ligation rats compared with sham groups

**Fig.4:**
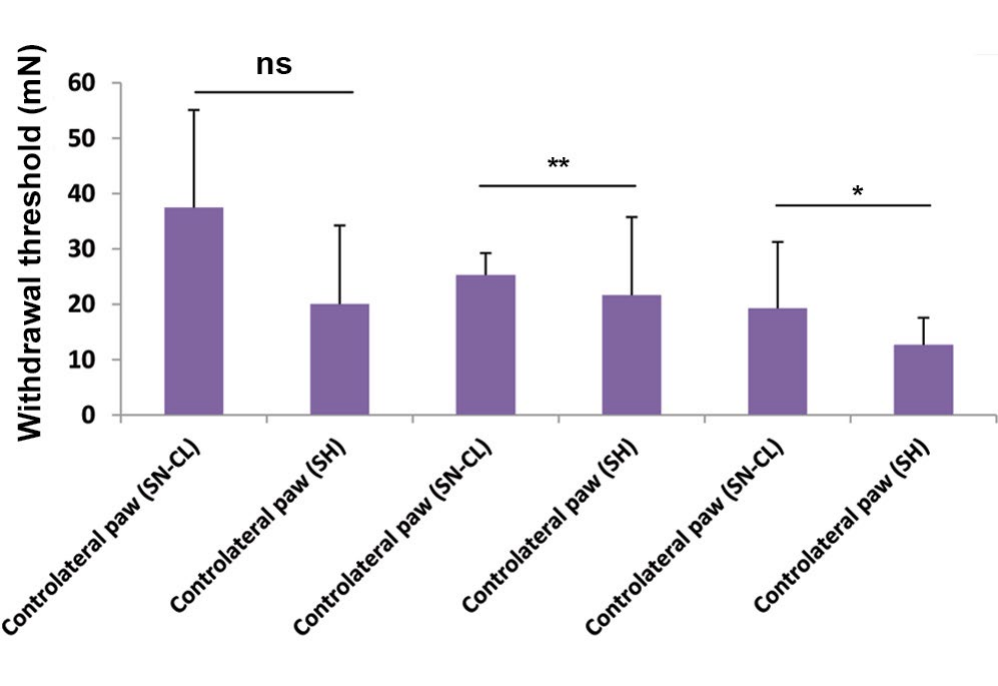
Graph showing compared mechanical contralateral allodynia in sciatic nerve chronic ligation with sham rats at 6^th^, 16^th^, and 26th days post- surgery (n=8 Wistar rats). Significant differences in paw withdrawal threshold are indicated by asterisks. ns; Non significant, *; P<0.05, and **; P<0.005.

Analysis of changes in sensory paw withdrawal thresholds showed a statistically difference between contralateral paw withdrawal thresholds in SN-CL and SH rats using Fisher’s exact test (P=0.0006), (P=0.003) and (P=0.03) at the 6^th^, 16^th^, and 26^th^ days after surgery, respectively (Fig.4).

### Hyperalgesia assessment in sciatic nerve chronic ligation and sham rats

**Fig.5:**
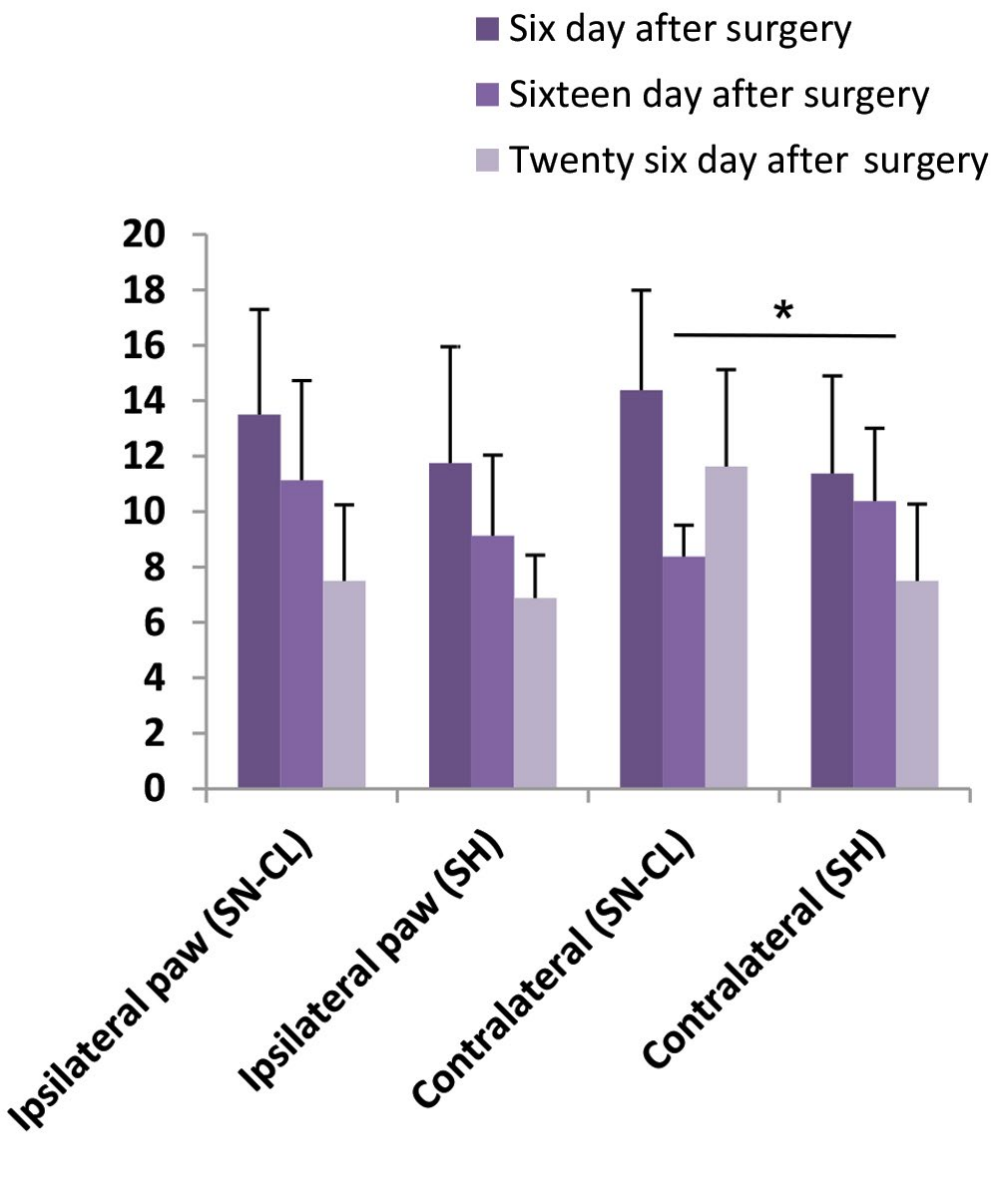
Assessment of mechanical hyperalgesia in sciatic nerve chronic ligation and sham groups at 6^th^, 16^th^, and 26^th^ days after surgery. (n=8 Wistar rats). Asterisks indicate the significant difference in paw withdrawal threshold. *; P<0.05.

Comparing tactile contralateral paw withdrawal thresholds mean in SN-CL rats (8.375 ± 1.133) with SH group ones (10.38 ± 2.632) revealed a significant difference (Fisher’s exact test, *P=0.0408, n=8) at the 16th-day post-surgery. However, at the 6th and the 26th days after surgery, the means were higher in SNCL group compared with SH one (14.38 ± 3.615 vs. 11.38 ± 3.520; 11.63 ± 3.495 vs 7.500 ± 2.771, n=8, respectively). For ipsilateral paw withdrawal threshold, the mean paw withdrawal thresholds in SNCL was increased at the 6th, 16th, and 26th days after surgery compared with SH rats (13.50 ± 3.794 vs. 11.75 ± 4.199, 11.125 vs. 9.125, 7.5 vs. 6.875, n=8 respectively) (Fig.5).

## Discussion

In this study, Von Frey monofilaments which are one of the most non-invasive techniques were used. vFFs are important tools for the study of mechanisms of cutaneous stimulation-induced sensory input (19, 20). To study peripheral neuropathic pain behavior, vFFs which have been exploited for the precise measurement of mechanosensitivity in most vertebrates in pain research were used (21-24). The filaments were applied at the mid-plantar surface just posterior to the footpads since this area is innervated mostly by terminal branches of the sciatic nerve. Also, applying hairs of different force is done to establish the paw withdrawal threshold (25).

The baseline withdrawal thresholds of each of the hind paws using von Frey hairs were determined for each rat prior to surgical manipulation (day 0). The, unilateral SN-CL of the left sciatic nerve was investigated to assess behavioral signs of peripheral neuropathic pain; however, it should be noted that many experimenters have performed on the right sciatic nerve, with similar pain and behavioral outcomes (26, 27). In this study, the behavioral evaluation was performed on different post-surgical days with an interval of ten days in SN-CL and SH rats in which the left sciatic nerve was exposed but not manipulated (28). On these days, the assessment of pain behavior was performed using Von Frey monofilaments (which are used in both preclinical and clinical assessment of allodynia). There is a limited amount of literature related to the time course measurement of mechanical allodynia using the SUDO method for measuring mechanical allodynia in SN-CL model of chronic neuropathic pain in contrast of the up-and-down method and the first response method for the reason that it was recently developed (29).

The assessment of ipsilateral tactile paw withdrawal thresholds showed that a transitory post-surgical mechanical allodynia was observed in the SH group from the 6th -day until 16th day post-surgery. In contrast, a longlasting ipsilateral mechanical allodynia was established in SN-CL until twenty-six days after surgery. In the SH group, transitory post-surgical contralateral allodynia was created until day 12 after surgery, in contrast to a longlasting ipsilateral allodynia until day 22 post-surgery, an assessment of contralateral allodynia was noted from day 22 to 26 post-surgery.

In a SN-CL model, data indicate that ipsilateral allodynia reached the day 6 after surgery and lasted until the 26^th^ day after surgery, the mechanical pain hypersensitivity was maximal at the 16^th^ -day when compared with ipsilateral mean paw withdrawal thresholds in sham-operated rats. In contrast, the contralateral allodynia appeared later on day 16 postoperatively in SN-CL animals.

At the 6^th^ -day after surgery, the absence of significant different determines the loss effect of sciatic nerve ligation, demonstrating that neuropathy was not leaded. In contrast, a very significant and significant statically differences, which observed in day 16 and 26 post-surgery, respectively showed that ipsilateral hind paw becomes more sensitive and gets more pain hypersensitivity at the sixteen-day, with a lower intensity of mechanical allodynia at the twenty-six-day post-operatively witch validate that SN-CL in rats seems to present significant quantitative changes proportional to the external stimulation in mechanical allodynia (30), demonstrated that in mice model of neuropathic pain, neuropathy developed from day 7 postoperatively and in most animals neuropathy, was still observed until day 21-27 post-operatively.

The primary outcome resulted from comparing ipsilateral hind paw withdrawal threshold in SN-CL rats with the SH group demonstrate that ipsilateral allodynia became more intensive on day 16 explained by the loss of sensations (31) resulted from sciatic nerve damage, the mechanical ipsilateral allodynia lasted until day 26 after surgery in SN-CL rats.

When comparing contralateral mean hind paw withdrawal thresholds of SN-CL rats with SH control ones, the significant statically differences showed that contralateral allodynia in SN-CL rats appeared on day 6 and lasted until day 26 postoperatively witch lead to an extraterritorial development of neuropathic signs (32).

For hyperalgesia assessment, data showed an absence of ipsilateral hyperalgesia development at the different days of mechanical paw withdrawal thresholds measurements with the development of contralateral hyperalgesia in SNCL rats just at the 16th day after surgery.

There are numerous problems with the use of patients or healthy volunteers in pain research. We can only use a modest stimulus that will not produce any irreversible harm, and we also have to take into account accompanying diseases, malingering, and the placebo effect. It is also very difficult to recruit significant numbers of patients needed for clinical trials. Therefore, pain research is often conducted using animal models. Examination of the pathogenesis of neuropathic pain has been accelerated by the introduction of rodent models of the nerve injury that produce behavior indicative of spontaneous and inducible pain.

We chose SN-CL as a chronic pain model that made a significant contribution in understanding the pathophysiological mechanisms in chronic pain, which is quite distinct from acute noxious pain. The model produces unilateral peripheral mononeuropathy, and it has been observed that symptoms in this rat model correspond to causalgia or complex regional pain syndrome in patients. It induces allodynia in rodents and other symptoms which are similar to those of neuropathic pain in humans.

More work is needed for determination of the most predictive animal models, removal of user bias and, an introduction of more complex outcome measures in behavioral tests. It is important to state that in pain research, the problem is even more pronounced due to the subjective nature of painful experience. Only humans can express and describe the emotional aspect of a painful experience.

## Conclusion

Sciatic nerve ligation induces a long-lasting of peripheral neuropathic pain signs with the absence of ipsilateral hyperalgesia development.

## References

[1] Michael B, Harald B, Troels SJ, Willem S, Olaitan S, Rolf-Detlef T, Johan AA, Giuliano A, José MB, Hanneke dB, Harald B, Tarun D, Nori G, Aleksandar J, Jürg K, Colin M, Anna M, Leonid P, Benedetto S, Shekhar S, Timothy JS (2006). Neurological disorders a public health approach. Neurological disorders: public health challenges.

[2] Treede RD (2018). The International Association for the Study of Pain definition of pain: as valid in 2018 as in 1979, but in need of regularly updated footnotes. Pain Rep.

[3] Merskey H, Bogduk N (1994). Classification of chronic pain: descriptions of chronic pain syndromes and definitions of pain terms.

[4] Michael B, Harald B, Troels SJ, Willem S, Olaitan S, Rolf-Detlef T, Johan AA, Giuliano A, José MB, Hanneke dB, Harald B, Tarun D (2006). Neurological disorders a public health approach. Neurological disorders: public health challenges.

[5] Laird B, Colvin L, Fallon M (2008). Management of cancer pain: basic principles and neuropathic cancer pain. Eur J Cancer.

[6] Bridges D, Thompson SW, Rice AS (2001). Mechanisms of neuropathic pain. Br J Anaesth.

[7] Woolf CJ, Ma Q (2007). Nociceptors--noxious stimulus detectors. Neuron.

[8] Abelson K, Roughan, John V (2011). Animal models in pain research.In: Hau J, Schapiro SJ, editors.Handbook of laboratory animal.3rd ed. New York: CRC Press.

[9] Lambert GA, Mallos G, Zagami AS (2009). Von Frey’s hairs--a review of their technology and use--a novel automated von Frey device for improved testing for hyperalgesia. J Neurosci Methods.

[10] Coyle DE, Sehlhorst CS, Mascari C (1995). Female rats are more susceptible to the development of neuropathic pain using the partial sciatic nerve ligation (PSNL) model. Neurosci Lett.

[11] Liu JH, Dacus AC (1989). Intramuscular injection of chlorpromazine decreases intraocular pressure by lowering systemic blood pressure. Curr Eye Res.

[12] Bennett GJ, Xie YK (1988). A peripheral mononeuropathy in rat that produces disorders of pain sensation like those seen in man. Pain.

[13] Bradman MJ, Ferrini F, Salio C, Merighi A (2015). Practical mechanical threshold estimation in rodents using von Frey hairs/SemmesWeinstein monofilaments: Towards a rational method. J Neurosci Methods.

[14] Victor Pires de Sousa M, Ferraresi C, Carolina de Magalhães A, Mateus Yoshimura E, Hamblin MR (2014). Building, testing and validating a set of home-made von Frey filaments: a precise, accurate and cost effective alternative for nociception assessment. J Neurosci Methods.

[15] Fruhstorfer H, Gross W, Selbmann O (2001). von Frey hairs: new materials for a new design. Eur J Pain.

[16] Mogil JS, Wilson SG, Wan Y, Kruger L (2001). Assessing nociception in murine subjects. Methods in pain research.

[17] Bonin RP, Bories C, De Koninck Y (2014). A simplified up-down method (SUDO) for measuring mechanical nociception in rodents using von Frey filaments. Mol Pain.

[18] Liu YT, Chen SD, Chuang YC, Shaw FZ (2017). Pregabalin, duloxetine, and diazepam selectively modulate acid-induced hyperalgesia and anxio-depressive comorbidity in rats. Neuropsychiatry.

[19] Chaplan SR, Bach FW, Pogrel JW, Chung JM, Yaksh TL (1994). Quantitative assessment of allodynia in the rat paw. J Neurosci Methods.

[20] Nirogi R, Goura V, Shanmuganathan D, Jayarajan P, Abraham R (2012). Comparison of manual and automated filaments for evaluation of neuropathic pain behavior in rats. J Pharmacol Toxicol Methods.

[21] Juszkiewicz-Donsbach J, Levy G (1962). Effect of small variations in heat stimulus temperature on the tail flick response of rats in analgesimetry. J Pharm Sci.

[22] Bonnet KA, Peterson KE (1975). Modification of the jump-flinch technique for measuring pain sensitivity in rats. Pharmacol Biochem Behav.

[23] Pitcher GM, Ritchie J, Henry JL (1999). Paw withdrawal threshold in the von Frey hair test is influenced by the surface on which the rat stands. J Neurosci Methods.

[24] Kim HT, Kim T, Novotny B, Khan N, Aksamit J, Siegel S (2014). Thermal hyperalgesia assessment for rats after spinal cord injury: developing valid and useful pain index. Spine J.

[25] Pitcher GM, Ritchie J, Henry JL (1999). Paw withdrawal threshold in the von Frey hair test is influenced by the surface on which the rat stands. J Neurosci Methods.

[26] Myers RR, Yamamoto T, Yaksh TL, Powell HC (1993). The role of focal nerve ischemia and Wallerian degeneration in peripheral nerve injury producing hyperesthesia. Anesthesiology.

[27] Grace PM, Hutchinson MR, Manavis J, Somogyi AA, Rolan PE (2010). A novel animal model of graded neuropathic pain: utility to investigate mechanisms of population heterogeneity. J Neurosci Methods.

[28] Polgár E, Hughes DI, Arham AZ, Todd AJ (2005). Loss of neurons from laminas I-III of the spinal dorsal horn is not required for development of tactile allodynia in the spared nerve injury model of neuropathic pain. J Neurosci.

[29] McMackin MZ, Lewin MR, Tabuena DR, Arreola FE, Moffatt C, Fuse M (2016). Use of von Frey filaments to assess nociceptive sensitization in the hornworm, Manduca sexta. J Neurosci Methods.

[30] van der Wal S, Cornelissen L, Behet M, Vaneker M, Steegers M, Vissers K (2015). Behavior of neuropathic pain in mice following chronic constriction injury comparing silk and catgut ligatures. Springerplus.

[31] Marchettini P (2006). Painful peripheral neuropathies. Curr Neuropharmacol.

[32] Pitcher GM, Ritchie J, Henry JL (1999). Nerve constriction in the rat: model of neuropathic, surgical and central pain. Pain.

